# Supplementary material for: Mapping the evolution of fertility support policies in China: A content and instrumental analysis
Source: PLoS One. 2025 Oct 9;20(10):e0332137. doi: 10.1371/journal.pone.0332137 (PMC12510515; doi:10.1371/journal.pone.0332137)
Supplement: S1 Appendix — (ZIP) [file pone.0332137.s001.zip › S1 Appendix. 226 original policy documents/16-国家卫生和计划生育委员会、财政部、国家中医药管理局关于做好2013年国家基本公共卫生服务项目工作的通知(FBM-CLI-4-205125).doc]

国家卫生和计划生育委员会、财政部、国家中医药管理局关于做好2013年国家基本公共卫生服务项目工作的通知

**发布部门：** [国家卫生和计划生育委员会(已撤销)](https://www.pkulaw.com/law/chl?Aggs.IssueDepartment=60319) [财政部](https://www.pkulaw.com/law/chl?Aggs.IssueDepartment=60210) [国家中医药管理局](https://www.pkulaw.com/law/chl?Aggs.IssueDepartment=7060306) [机构沿革](javascript:void(0))

**发文字号：**卫计生发〔2013〕26号

**发布日期：**2013.06.06

**实施日期：**2013.06.06

**时效性：** [现行有效](https://www.pkulaw.com/law/chl?Aggs.TimelinessDic=01)

**效力级别：** [部门工作文件](https://www.pkulaw.com/law/chl?Aggs.EffectivenessDic=XE0303)

**法规类别：** [卫生综合规定](https://www.pkulaw.com/law/chl?Aggs.Category=09601)

国家卫生和计划生育委员会、财政部、国家中医药管理局关于做好2013年国家基本公共卫生服务项目工作的通知
（卫计生发〔2013〕26号）

各省、自治区、直辖市卫生厅局（卫生计生委）、人口计生委、财政厅局、中医药管理局，新疆生产建设兵团卫生局、人口计生委、财务局：
　　为贯彻落实国务院《“十二五”期间深化医药卫生体制改革规划暨实施方案》有关精神，做好2013年国家基本公共卫生服务项目工作，现将有关事项通知如下：
　　**一、**提高人均基本公共卫生服务经费补助标准
　　2013年人均基本公共卫生服务经费补助标准由25元提高至30元。新增经费主要用于做实、做细、做深现有基本公共卫生服务，同时进一步扩大受益人群范围，强化基础性服务项目。一是用于扩大建立居民电子健康档案、高血压和糖尿病患者健康管理、老年人健康管理覆盖面；二是适当提高预防接种、重性精神疾病患者健康管理、传染病及突发公共卫生事件报告和处理、卫生监督协管等服务项目补助水平；三是将中医药健康管理服务纳入基本公共卫生服务范围。各地要结合本地区实际情况，科学测算各项服务的补偿水平，合理确定工作任务目标，要以提高基本公共卫生服务质量为重点，进一步健全项目管理制度，强化绩效考核，改进服务方式，提高服务水平，切实保障群众受益。

　　**二、**主要工作任务
　　（一）提高健康档案使用率。以慢性病患者、老年人、孕产妇、儿童以及基层医疗卫生机构就诊人群等为重点，扩大健康档案覆盖面，2013年居民健康档案全国总体建档率累计要达到80%以上，其中电子建档率要达到65%以上。要进一步规范健康档案信息采集，加大检查力度，保证档案信息真实完整。各地要加快基层医疗卫生机构信息系统建设，条件成熟的地区要同步推进居民健康卡发放和使用，促进健康档案与基层医疗卫生服务有效衔接，建立居民健康档案动态更新机制，提高健康档案使用率。
　　（二）丰富健康教育内容和形式。各地要树立健康优先、健康教育先行理念，将健康教育融入各项基本公共卫生服务中。丰富基层医疗卫生机构健康教育内容和形式，提高健康教育工作科学性和适用性，注重发挥中医药健康教育作用。结合爱国卫生工作和各种卫生主题宣传日，进一步利用网络、短信等形式，开展面向公众的健康教育。加强个体化健康指导，为重点人群制订适宜的健康教育方案，提高服务对象参与度和依从性。
　　（三）巩固和加强预防接种工作。2013年，各地以乡镇（街道）为单位，将适龄儿童（包括流动人口）国家免疫规划疫苗接种率保持在90%以上。要进一步改善接种服务环境，强化安全注射，有效处置疑似预防接种异常反应。要合理布设接种点，提高预防接种可及性。合理安排接种工作日，实行多种方式和分时段预约接种，将预防接种与儿童保健有机结合。加强预防接种信息管理，加大流动人口预防接种力度，定期开展漏种排查并及时补种。
　　（四）提高儿童健康管理水平。各地妇幼保健机构要及时汇总本地新生儿出生信息，并转至基层医疗卫生机构，方便其开展新生儿家庭访视。要按照《[国家基本公共卫生服务规范（2011年版）](https://www.pkulaw.com/chl/aa2f3b126e36e49cbdfb.html)》要求，在规定时间内，按照相应年龄，为辖区内7岁以下儿童提供体格检查、血常规检测、生长发育和心理行为评估、健康指导等服务。以县（区、市）为单位，2013年7岁以下儿童健康管理率要达到80%以上。
　　（五）加强孕产妇健康管理。各地要积极为基层医疗卫生机构创造条件开展孕产妇健康管理服务，按规范要求为孕产妇提供保健服务，保证孕产妇至少接受5次产前检查和2次产后访视服务。暂不具备条件的地区，可由县级卫生行政部门通过购买服务方式由辖区内其他有资质医疗卫生机构提供孕产妇健康管理服务。以县（区、市）为单位，2013年孕产妇系统管理率要达到80%以上。
　　（六）做好老年人健康体检工作。基层医疗卫生机构要根据辖区65岁以上老年人数量和身体状况，制订年度计划，合理安排医务人员和工作时间，采取多种形式，有序做好老年人健康体检工作。要保证老年人必要的体格检查和实验室检查项目，并做好检查结果反馈工作，切实发挥体检在疾病筛查和健康指导中的作用，提高老年人健康保健意识。以县（区、市）为单位，2013年65岁以上老年人健康管理率要达到65%以上。
　　（七）提高慢性病管理率和控制率。通过日常门诊、健康体检、建立健康档案等途径，加大高血压、糖尿病患者筛查和发现力度，准确掌握患病情况，提高知晓率。要及时将发现的患者纳入健康管理，并规范提供服务，2013年，以县（区、市）为单位，高血压和糖尿病患者健康管理率要分别达到35%和20%以上，全国管理人数分别达到7000万人和2000万人以上。要将慢性病随访工作与基层医疗卫生机构门诊服务相结合，根据患者病情，加强生活方式和用药指导，提高患者依从性和血压、血糖控制率。
　　（八）加强重性精神疾病患者管理。地方各级卫生计生行政部门要加强与公安、民政、残联等有关部门协作，加强与街道（乡镇）、居（村）委会联系，建立日常筛查机制，及时发现重性精神疾病患者。要及时为新发现患者建立健康档案并及时更新，做到发现一例、管理一例。加强分类管理，根据患者病情相应增加随访次数，指导患者科学用药，提高病情稳定率。2013年，要按照“应管尽管”原则，将发现并登记在册的居家治疗重性精神疾病患者在知情同意的基础上全部纳入管理。
　　（九）做好传染病和突发公共卫生事件报告和处理工作。基层医疗卫生机构要按照规范要求，加强传染病疫情和突发公共卫生事件风险排查，做好发现、登记等工作，并按照相关时限及时上报。积极协助有关部门做好病人医疗救治、流行病学调查、疫点疫区处理、应急接种和预防性服药等工作。
　　（十）积极推进中医药健康管理服务。进一步发挥中医药在基本公共卫生服务中的作用，2013年起开展老年人中医体质辨识和儿童中医调养服务。2013年，各省（区、市）中医药健康管理服务目标人群覆盖率要达到30%。各地要加强基层医疗卫生机构中医药服务能力建设，合理配置中医人员，加强人员培训。国家卫生计生委、国家中医药局将另行制订中医药健康管理服务规范。
　　（十一）完善卫生监督协管服务。2013年以县（区、市）为单位，政府办基层医疗卫生机构开展卫生监督协管服务的比例要达到90%以上。完善卫生监督协管制度，规范工作流程。卫生监督机构要加强对基层医疗卫生机构的指导，提高其开展卫生监督协管工作的规范性和有效性，认真开展巡查、信息收集和报告等工作。
　　地方各级卫生计生部门要积极探索建立基本公共卫生服务和计划生育技术服务衔接机制，加强基层医疗卫生机构和计划生育技术服务机构分工合作，逐步将计划生育咨询指导、优生优育健康知识宣传等服务融入健康教育、孕产妇健康管理等服务中，进一步提高居民获得基本公共卫生服务和计划生育技术服务的可及性和方便性。

　　**三、**规范资金管理
　　2013年中央将继续对各地给予补助，地方各级财政部门要足额安排补助资金预算。省级财政部门要统筹使用中央补助资金，加大对困难地区支持力度。要完善资金拨付方式，采取“先预拨、后结算”等方式，进一步加快资金拨付进度，提高资金使用效率。加大资金监管力度，确保专款专用，严禁截留和挪用。县级卫生行政部门要会同财政部门合理测算确定各项服务成本补偿标准，并根据基层医疗卫生机构提供的服务数量、质量和绩效考核结果安排和拨付资金，不得简单地按照基层医疗卫生机构人员和支出水平核拨资金。

　　**四、**加强绩效考核和日常管理
　　各地要健全绩效考核机制，完善考核方案，细化考核内容，合理设定考核指标，开展基本公共卫生服务项目考核，并将考核结果与人员管理和资金分配挂钩。鼓励探索实行第三方考核。2013年，国家卫生计生委、财政部将对各省（区、市）2012年度基本公共卫生服务项目实施情况进行考核。地方各级卫生计生行政部门要加强对基本公共卫生服务项目的管理，进一步健全管理组织，完善系统内部各业务部门协作机制。要根据当地实际，合理分解各项任务，明确项目实施单位，加强项目日常管理和督导检查，确保各项服务规范开展和工作任务的落实。要加强服务工作量数据上报和管理，建立数据月报或季报制度，并以适当形式公布。要坚决制止虚报浮夸行为，一经发现，要通过通报批评、取消服务资格、扣减经费补助等方式予以严肃处理。各地要进一步加大宣传力度，通过报纸、广播、电视等，宣传基本公共卫生服务项目，提高居民知晓率。基层医疗卫生机构要在辖区显著位置以适当形式公示基本公共卫生服务项目免费政策、服务对象、服务内容、服务数量和服务方式等，接受居民和媒体监督。要及时发现并协调解决项目实施中的问题，深入总结经验，通过多种形式加强经验交流。
　　附件：国家基本公共卫生服务项目一览表（2013年）

国家卫生和计划生育委员会
财政部
国家中医药管理局
2013年6月6日

　　附件
　　国家基本公共卫生服务项目一览表（2013年）

| 序 号 | 类 别 | 服务对象 | 项目及内容 |
| --- | --- | --- | --- |
| 一 | 建立居民健康档案 | 辖区内常住居民，包括居住半年以上非户籍居民 | 1.建立健康档案 |
|  |  |  | 2.健康档案维护管理 |
| 二 | 健康教育 | 辖区内居民 | 1.提供健康教育资料 |
|  |  |  | 2.设置健康教育宣传栏 |
|  |  |  | 3.开展公众健康咨询服务 |
|  |  |  | 4.举办健康知识讲座 |
|  |  |  | 5.开展个体化健康教育 |
| 三 | 预防接种* | 辖区内0～6岁儿童和其他重点人群 | 1.预防接种管理 |
|  |  |  | 2.预防接种 |
|  |  |  | 3.疑似预防接种异常反应处理 |
| 四 | 儿童健康管理 | 辖区内居住的0～6岁儿童 | 1.新生儿家庭访视 |
|  |  |  | 2.新生儿满月健康管理 |
|  |  |  | 3.婴幼儿健康管理 |
|  |  |  | 4.学龄前儿童健康管理 |
| 五 | 孕产妇健康管理 | 辖区内居住的孕产妇 | 1.孕早期健康管理 |
|  |  |  | 2.孕中期健康管理 |
|  |  |  | 3.孕晚期健康管理 |
|  |  |  | 4.产后访视 |
|  |  |  | 5.产后42天健康检查 |
| 六 | 老年人健康管理 | 辖区内65岁及以上常住居民 | 1.生活方式和健康状况评估 |
|  |  |  | 2.体格检查 |
|  |  |  | 3.辅助检查 |
|  |  |  | 4.健康指导 |
| 七 | 慢性病患者健康管理（高血压） | 辖区内35岁及以上原发性高血压患者 | 1.筛查 |
|  |  |  | 2.随访评估和分类干预 |
|  |  |  | 3.健康体检 |
|  | 慢性病患者健康管理（2型糖尿病） | 辖区内35岁及以上2型糖尿病患者 | 1.筛查 |
|  |  |  | 2.随访评估和分类干预 |
|  |  |  | 3.健康体检 |
| 八 | 重性精神疾病患者管理* | 辖区内诊断明确、在家居住的重性精神疾病患者 | 1.重性精神疾病患者信息管理 |
|  |  |  | 2.随访评估和分类干预 |
|  |  |  | 3.健康体检 |
| 九 | 传染病和突发公共卫生事件报告和处理* | 辖区内服务人口 | 1.传染病疫情和突发公共卫生事件风险管理 |
|  |  |  | 2.传染病和突发公共卫生事件的发现和登记 |
|  |  |  | 3.传染病和突发公共卫生事件相关信息报告 |
|  |  |  | 4.传染病和突发公共卫生事件的处理 |
| 十 | 中医药健康管理& | 辖区内65岁及以上常住居民和0～36个月儿童 | 1.老年人中医体质辨识 |
|  |  |  | 2.儿童中医调养 |
| 十一 | 卫生监督协管* | 辖区内居民 | 1.食品安全信息报告 |
|  |  |  | 2.职业卫生咨询指导 |
|  |  |  | 3.饮用水卫生安全巡查 |
|  |  |  | 4.学校卫生服务 |
|  |  |  | 5.非法行医和非法采供血信息报告 |

　　注：2013年起，人均基本公共卫生服务经费由25元增加到30元。表示新增经费用于提高服务覆盖面的项目；*表示新增经费用于提高服务保障水平的项目；&表示新增加的服务类别。

© 北大法宝：（[www.pkulaw.com](https://www.pkulaw.com/)) 专业提供法律信息、法学知识和法律软件领域各类解决方案。北大法宝为您提供丰富的参考资料，正式引用法规条文时请与标准文本核对。
欢迎查看所有[产品和服务](https://www.pkulaw.com/helps/2.html)。法宝快讯：[如何快速找到您需要的检索结果？](https://www.pkulaw.com/helps/70.html)[法宝](https://www.pkulaw.com/helps/69.html)V6有何新特色？

扫描二维码阅读原文

**原文链接：**<https://www.pkulaw.com/chl/e8fa9e63788e1c42bdfb.html>
